# Ethical imperatives of timely access to orphan drugs: is possible to reconcile economic incentives and patients’ health needs?

**DOI:** 10.1186/s13023-016-0551-7

**Published:** 2017-01-05

**Authors:** R. Rodriguez-Monguio, T. Spargo, E. Seoane-Vazquez

**Affiliations:** 1 Department of Health Promotion and Policy, School of Public Health and Health Sciences, University of Massachusetts, 322 Arnold House. 715 North Pleasant Street, Amherst, MA 01003-9304 USA; 2 Department of Health Promotion and Policy, School of Public Health and Health Sciences, University of Massachusetts, Amherst, 325 Arnold House. 715 North Pleasant Street, Amherst, MA 01003-9304 USA; 3 Department of Pharmaceutical Business and Administrative Sciences Massachusetts College of Pharmacy and Health Sciences, MCPHS University, Room W324 179 Longwood Ave, Boston, MA 02115-5804 USA; 4 International Center for Pharmaceutical Economics and Policy Massachusetts College of Pharmacy and Health Sciences, MCPHS University, Room W324 179 Longwood Ave, Boston, MA 02115-5804 USA; 5 Division of General Medicine and Primary Care, Brigham and Women’s Hospital, Boston, MA USA

**Keywords:** Orphan drugs, Rare diseases, Orphan diseases, Research and development, FDA, Economic incentives, Ethical aspects

## Abstract

**Background:**

More than 6,800 rare diseases and conditions have been identified in the US, which affect 25–30 million Americans. In 1983, the US Congress enacted the Orphan Drug Act (ODA) to encourage the development and marketing of drugs to treat rare diseases and conditions. This study analyzed all orphan designations and FDA approvals since 1983 through 2015, discussed the effectiveness of incentives for the development of treatments for rare diseases, and reflected on the ethical imperatives for timely access to orphan drugs.

**Methods:**

Study data were derived from the Food and Drug Administration (FDA) Orange Book and the Office of Orphan Drugs Development. A search was conducted to assess literature on the ethical principles and economic incentives for the development of orphan drugs.

**Results:**

In the period 1983–2015, the FDA granted 3,647 orphan drug designations and 554 orphan drug approvals. The orphan drug approvals corresponded to 438 different brand names. Cancer was the therapeutic area with the highest number of approvals. The increased number of patients with rare diseases and the growth in the cost of orphan drugs pose a significant economic burden for patients, public programs and private third party payers. Regulatory differences to qualify for orphan designation and various population thresholds employed by the FDA and the European Medicines Agency lead to further unmet health needs for patients with rare diseases and aggravate health inequities. There is no societal consensus on the population and economic thresholds, the drug effectiveness indicator(s), or the societal value to be placed for the approval and reimbursement of orphan drugs.

**Conclusion:**

Orphan drug development and marketing in the US concentrate in few therapeutic areas. Despite the increase in the number of FDA approved orphan drugs, the unmet needs of patients with rare diseases evidence that the current incentives are not efficiently stimulating orphan drug development. There is need to balance economic incentives to stimulate the development and marketing of orphan drugs without jeopardizing patients’ access to treatment. Thus, aligning pharmaceutical companies’ incentives with societal budgetary constraints is necessary and the ethical imperatives of timely access to orphan drugs need to be agreed upon.

## Background

Chronic conditions such as heart disease, stroke, cancer, diabetes, and arthritis affect millions of patients in the United States (US) [1]. These prevalent diseases have traditionally attracted significant research and economic resources from public and private institutions, organizations and pharmaceutical companies. Simultaneously, a large number of diseases and conditions afflict a relatively small number of patients. As of March 18, 2016, the National Institutes of Health Genetic and Rare Disease Information Center have listed more than 6,800 rare diseases and conditions in the US, which affect an estimated 25–30 million Americans [2, 3]. Approximately, 250 new rare diseases and conditions are described each year [4]. Growing attention has been given to ultra-rare diseases (i.e., disease or condition that affects a small number of patients in the US), [5–8]. Although there is no consensus yet on the definition of an ultra-rare disease, the concept has been applied in the literature to diseases that have a prevalence of <1 per 50,000 persons [9].

Most modern societies consider an ethical imperative to ensure patients’ access to drugs for the prevention and treatment of severe and life-threatening diseases. Historically, research and development (R&D) efforts focused on the most prevalent diseases and conditions. Only recently, attention has turned to a growing number of rare diseases that affect a large combined number of patients and require new and improved treatment alternatives for addressing patients’ unmet health needs.

Regulatory initiatives and R&D efforts during the past three decades resulted in the development and approval of a significant number of drugs for rare diseases and conditions (i.e. orphan drugs). While orphan drugs improve the health status and quality of life of patients, the cost of new orphan drugs also limit patients’ access to treatment.

This study analyzed all orphan designations and US Food and Drug Administration (FDA) approvals since the enactment of the Orphan Drug Act (ODA) in 1983 through December 31, 2015, discussed the effectiveness of incentives for development and marketing of treatments for rare diseases and conditions and patients’ barriers to access orphan drugs, and reflected on the ethical imperatives for timely access to orphan drugs in the current context of its societal value.

In spite of the significant increase in the number of orphan drugs approved by the FDA since the enactment of the ODA in 1983 and the faster than the economy increases in orphan drug prices, the economics of the orphan drug market remain controversial among some scholars and stakeholders. Given the growing number of rare and ultra-rare diseases, the overall rising cost of healthcare and stretched budgets to cover rare disease treatment, this study has the potential to enrich and appraise the current debate on the ethical imperatives of providing timely access to orphan drugs. A comprehensive understanding of the ethical considerations of access to safe and effective orphan drugs is essential as research continues related to the regulation of drug development for rare diseases and to the implementation of public policies that may impact treatment affordability.

## Methods

Study data were derived from the FDA Approved Drug Products with Therapeutic Equivalence Evaluations (i.e. Orange Book-OB) versions from 1983–2015, the electronic version of the OB, the FDA OOPD List of Orphan Designations and Approvals, documents and data from the FDA's website. Data were updated through December 31, 2015. Study data included all orphan designations and approvals/licenses listed by the FDA since the enactment of the ODA in 1983 through December 31, 2015.

A PubMed and EconLit search of peer-reviewed papers on the incentives for the development of orphan drugs and the ethical dilemmas to be considered when funding research and treatment for rare diseases and conditions was conducted using the search terms “orphan drugs” OR “rare diseases” AND "access" OR "price" OR "cost" OR "incentives" OR "ethics", with limits for English language, publication from 1983–2016. Our search defined, a priori, a set of criteria for selecting studies, assessing the methodologic quality of those studies, and synthesizing the evidence across studies. Criteria for inclusion or exclusion of retrieved articles were determined before the literature search; article review was conducted independently by 2 reviewers. Manuscripts were excluded if they were: 1) not published in English or as full-length peer-reviewed manuscripts (e.g. letters to the editor, commentary or point of view), 2) primarily assessed safety or effectiveness of orphan drugs, 3) duplicate articles, 4) focused on societal willingness to pay, 5) case studies for particular diseases or 6) updated in a more recent article. References cited in the retained articles were reviewed for additional articles. Overall, of 262 articles retrieved, 24 articles were retained. Data were qualitatively assessed following a structured and standardized approach. A standardized data abstraction form, using a spreadsheet template in excel, and a checklist were developed and utilized by the authors to assess retrieved manuscripts. The data abstraction form included study question, study design and characteristics of studies including year when the study was conducted and country, study population, sample size, patient demographics and assessed clinical condition(s), perspective of the analysis, and study findings. Two authors abstracted information from studies independently. The results from the data abstraction were compared only after completing the review of the articles. Discrepancies between authors were resolved by the third author.

## Results

### Patients’ unmet health needs

The lack of clinical alternatives for the prevention and treatment of rare diseases and conditions has been attributed to the difficulty of recovering the R&D cost due to the small size of the population and potential for profits. In response to those concerns, in 1983, the US Congress enacted the Orphan Drug Act (ODA) to encourage the development and marketing of drugs to treat rare diseases and conditions [10]. Orphan drug status initially applied to products whose sales in the US market would not cover the costs incurred during product development. In 1984, the ODA amendment expanded the definition of orphan drugs to include drugs for any disease or condition that affect less than 200,000 persons in the US [11].

The US and the European Union (EU) use different methods and population thresholds to determine if a drug qualifies for orphan designation. The US Food and Drugs Administration (FDA) uses the prevalence of the disease (i.e. number of people that have the disease), while the European Medicines Agency (EMA) uses the prevalence proportion (i.e. proportion of people in the population that have the disease). In the EU, the prevalence of the condition must not be more than 5 in 10,000 persons [12]. Employing the disease prevalence instead of a disease prevalence proportion means that as the population grows, a smaller percentage of the population will need to be impacted by a rare disease in order for a drug to qualify for the orphan drug legal provisions. On January 1, 1984 a drug for a disease affecting less than 1 in 1,174 Americans would had qualified for orphan designation based on the 200,000 patient population threshold - authors’ estimations using data from the US Census Bureau, 2016. On January 1, 2016, a disease must affect less than 1 in 1,615 Americans to qualify for such orphan designation. Therefore, the use of disease prevalence conflicts with the overall concept of vertical justice of health as the regulatory arbitrary cut-off point will continue to proportionally exclude rarer diseases and conditions over time. Conversely, it is expected that a growing population will lead to the identification of more rare diseases and thus, additional health care needs will have to be addressed with fewer resources for each condition.

**Fig. 1:**
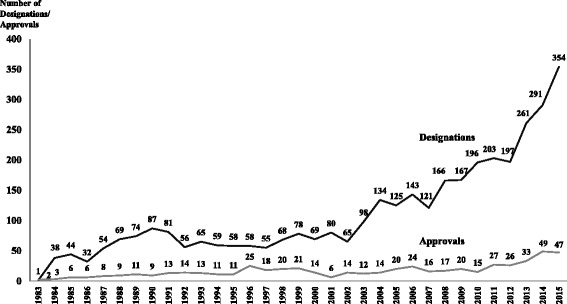
FDA Orphan Drug Designations and Approvals, 1983–2015

The ODA has been credited to result in a significant increase in the number of drug approvals for rare diseases and conditions [13, 14]. In the period 1983–2015, the FDA granted 3,647 orphan drug designations and 554 orphan drug approvals (Fig. 1). The orphan drug approvals correspond to 438 different brand names; 53 branded drugs had more than 1 orphan approval (range 2–6). The orphan drug approvals targeted 277 rare diseases. There were 191 diseases with 1 orphan designation and 86 diseases with two or more orphan designations. Cancer was the therapeutic area with the highest number of approvals (177 orphan approvals, 31.9% of the total number of orphan approvals), followed by infectious diseases excluding HIV (46, 8.3%), hemophilia and other bleeding disorders (32, 5.8%), HIV and related comorbidities (19, 3.4%), growth failure (13, 2.3%), pulmonary arterial hypertension (12, 2.2%), and transplant related designations (11, 2.0%). Other therapeutic classes had 244 orphan approvals representing 44.0% of the total approvals [15]. Individuals living with rare diseases often experience delays in diagnosis, misdiagnosis, and psychological and economic stress [16]. Physicians may lack needed clinical knowledge about rare diseases and available treatments leading to delayed diagnosis and inadequate care. In a survey of 5,980 patients and patient caregivers living in 17 European countries, assessing eight prevalent rare diseases, Kole & Faurisson found that 41% of orphan disease patients received at least one incorrect diagnosis prior to receiving a proper diagnosis [16]. Furthermore, patient’s care mainly focuses on managing symptoms instead of holistically addressing the patient health care needs.

### Economic incentives for the development and marketing of orphan drugs

Under the provisions of the ODA (1983) economic incentives to develop and market orphan drugs, include grant funding for academic-based researchers or companies, tax credits for expenditures incurred during the clinical trials phase, waivers of the Prescription Drug User Fee Act (1992) filing fees, and a seven-year market exclusivity for FDA-designated orphan drug indications. During the orphan market exclusivity period, the FDA cannot approve a new drug application or a generic drug application for the same product and for the same rare disease indication. In the EU, the European Parliament adopted the Orphan Drug Regulation 141/2000 [10] that provides similar market incentives to encourage development of orphan drugs.

There is a risk that developing orphan drugs for low prevalence rare diseases and consequently small market size is less attractive for the pharmaceutical industry [6]. Prior research found a modest impact of the ODA seven-year orphan drug market exclusivity provisions. The orphan drug market exclusivity provision increased the effective patent life of orphan new molecular entities (i.e., a new drug containing an active ingredient that has never before been approved for marketing in the US) by an average of 0.8 years [17]. Nevertheless, orphan drugs represent an increasingly important component of the pharmaceutical market. The orphan drug market represented approximately 22% of total pharmaceutical sales in 2010 with a mean per year economic value per drug of US$637 million [18]. The return of the investment in an orphan drug exceeds the return of the investment in a non-orphan drug (8.4% and 2.3%, respectively) [19]. The excess in the return of the investment is even greater when the drugs are brought to the market (30.1% and 17.1% for orphan and non-orphan drugs, respectively) [19]. Furthermore, orphan drugs can bring in significant revenues for pharmaceutical companies in global sales, [20] raising criticisms of high treatment costs of orphan drug products [21] and challenging the assumption that developing orphan drugs without public support is not profitable for pharmaceutical companies.

Current incentives to develop and market orphan drugs do not promote long-term innovation [22] or address some of the pressing concerns regarding treatment affordability for patients and third party payers [23]. In addition, patients may end up paying twice for the same drug through public funds to develop orphan drugs and the cost of treatment [17]. Therefore, there is need to balance economic incentives to stimulate development and marketing of orphan drugs without threating affordability. Despite the significant increase in the number of FDA approved orphan drugs during the last three decades, the unmet needs of rare disease patients suggest that the current financial incentives are not efficiently stimulating orphan drug development and marketing [17, 24].

### Barriers to access treatment for rare diseases

The cost of care for rare diseases poses an important economic burden to patients, third party payers and the society at large. The average annual direct health care cost per patient with rare diseases varies significantly by patient and disease characteristics [25]. In general, the cost of orphan drugs exceeds the cost paid for drugs for common diseases and the standard thresholds used to determine the cost-effectiveness of drugs [26]. In the US, estimations of the average annual direct health care cost per patient range from $118,293 (2005 USD) to $161,441 (2003 USD) for hemophilia [27], $63,127 (2006 USD) for cystic fibrosis [28], and $28,590 (2012 USD) for the Duchenne muscular dystrophy [29]. Inpatient care and prescription drugs are the main health care cost components related to rare diseases treatment cost.

Concerns have been raised about the high prices of orphan drugs [13, 18, 20, 30]. The price of orphan drugs is often the most significant barrier for patients to access care. Life-threatening health conditions and the lack of therapeutic alternatives creates an inelastic demand for orphan drugs leading to high prices in a market with already limited competition. The current reimbursement of orphan drugs on an exceptional basis may not be economically sustainable due to the existing budget constraints and the growing number of orphan drugs approved by the FDA [26].

### The societal value of orphan drugs

The societal value of orphan drugs can be framed by a variety of ethical constructs. If society wishes to maximize total net benefits, in line with a classic utilitarian doctrine (i.e., maximize utility for the greatest number of individuals in society), the cost-benefit ratio of rare diseases may be less favorable to receive public funding as they would in an egalitarian approach (i.e., maximize equality of individuals) [31]. The goal of achieving an egalitarian outcome can be based on reaching a predetermined threshold of health, a general prioritization of the worst off, or amount of resources for an individual [31]. Thus, an egalitarian doctrine would provide a greater foundation for public funding for orphan drugs development and treatment coverage for rare diseases. Alternatively, the rule of rescue, or the ability to intervene if a treatment becomes available, may also provide a justification for funding rare disease treatments with public resources. This argument presupposes that rare disease patients will have timely access and will be positively impacted by these treatments [32].

The ethics of the resource allocation to funding orphan drugs has been discussed but not agreed upon in the literature [22, 33–35]. There is no societal consensus related to whether or not the size of the patient population, in itself, is a justifiable factor in employing distinct measures of effectiveness and economic evaluation (e.g. cost-effectiveness) for the approval and reimbursement of orphan drugs. While some authors argue that the special status argument for public funding and reimbursement of orphan drugs does not stand up to critical assessment [34], others have strengthened the importance of using economic evaluation, political debate [23], and social dialogue [23, 35] to balance distributive justice to orphan drug development and access. Further, the outcome measure of effectiveness, whether premature mortality, quality adjusted life years, achieving a minimum acceptable life expectancy, or any other proposed metrics remains unclear [22, 34].

The average treatment cost is often skewed by a small proportion of patients requiring a large amount of health care resources [25, 36]. In addition, making inferences about the average cost per patient or per rare disease is challenging due to the high-degree of variability in the health care needs, and the type and cost of treatment. These considerations, may also vary on an individual level, at which point the argument is no longer looking at a potentially favored group, but prioritizing one patient over another, which runs counter to egalitarian concepts [34].

The role of identifiability of the patient population is also a component of the rule of rescue when making decisions about allocation of resources to rare diseases [31]. Patient registries and genetic screening, when available, are becoming increasingly more common to help identify patients. Patient registries provide valuable but also sensitive data, and thus, require patient privacy protections and a thorough understanding of the ethical and legal implications of proprietary use of medical data. All orphan drug patient registries are subject to federal privacy protections in the US. However, the rare disease population may be more vulnerable when it comes to protecting patient privacy as the small population affected, and often distinctive characteristics of the conditions, make potentially easier to identify individuals [37]. In addition, data ownership becomes challenging for rare diseases as the partnership models for registries are often more complex and the data have the potential to lead to more profitable treatments. Efforts have been led by government agencies, patient organizations, and independent health organizations in the US and Europe to outline best practices for patient registries [37].

### Ethical imperatives for timely access to orphan drugs

Orphan drugs are often for treatment of life-threatening diseases. In this context, the right to life may be constructed as the right to health. The Universal Declaration of Human Rights, proclaimed by the General Assembly of the United Nations in 1948 established the right to life codified in the European Convention on Human Rights (1950) [38]. Constitutional rights in western countries often include the right to life as one of the fundamental moral principles. The right to the “highest attainable standard of health” was first depicted in 1946 in the Constitution of the World Health Organization [39]. Since then, the right to health has been included in several international treaties and declarations and it is protected constitutionally in most developed economies with the notable exception of the US [40]. The European Court of Human Rights (ECHR) has interpreted the right to life as protecting the need for medical care [38].

The ECHR addressed the need for orphan drug treatment funding in Nitecki vs. Poland (2002) [41]. Nitecki, a Polish National with amyotrophic lateral sclerosis, challenged Poland’s refusal to refund the full cost of his drug under his right to life under Article 2 of the Convention of Human Rights. The ECHR found that the case was not a violation of the Convention of Human Rights, although the ECHR asserted this was due to the government’s funding already more than two-thirds of the treatment cost.

Rare diseases often result in disability. The Convention on the Rights of Persons with Disabilities, adopted by the United Nations (UN) in 2006, echoed and expanded upon the ideas articulated in the Universal Declaration of Human Rights (1950) by recognizing the right to enjoy “the highest attainable standard of health without discrimination on the basis of disability” [42]. The Universal Declaration of Human Rights afforded all of the rights and freedoms “without distinction of any kind, such as race, color, sex, language, religion, political or other opinion, national or social origin, property, birth or other status.” Nevertheless, protections for people with disabilities was not fully articulated until the Convention on the Rights of Persons with Disabilities in 2006.

In 2009, the Council of the European Union (EU) approved the conclusions of the Convention on the Rights of Persons with Disabilities (2006) and it was ratified in 2011 [43]. EU country members also enacted their own regulations related to disability. Although, the US signed the Convention in 2009, Congress has yet to ratify the document [43]. In the US, the most relevant disability legislation is the Americans with Disabilities Act (ADA) of 1990 [44]. The ADA definition of a person with a disability is “a person who has a physical or mental impairment that substantially limits one or more major life activities, a person who has a history or record of such an impairment, or a person who is perceived by others as having such an impairment” [44]. Further, individuals with severe rare diseases may qualify for additional protections and access to care under the US Social Security Act [45]. Neither of these regulations or their subsequent amendments list the conditions that are considered disabilities.

In the US, the Patient Protection and Affordable Care Act of 2010 implemented comprehensive health insurance reforms to improve health care coverage [46]. Although, rare diseases and orphan products are explicitly mentioned in the law, in the context of clinical trials and orphan product exclusions from other pharmaceutical sales regulation, the coverage of orphan drugs is not addressed in the Act.

Country specific court cases have led to a broad interpretation of national legislation in support of patient’s rights to health care [47] including treatment when no therapeutic alternative exist. Court cases may support patients’ rights to health and health care in cases where clear guidelines are not codified by law and individuals are denied access to treatment. The judicial review approach focuses on challenging regulations, policies and administrative decisions related to specific patient cases that may be considered violations of the constitutional and legal framework. In *R (on the application of Rogers)* vs. *Swindon NHS Primary Care Trust and another* (2006) [48], for example, the England and Wales Court of Appeals found that while it would be rational to deny treatment to a lone individual, denying treatment to patients, who could all potentially benefit from the drug, was irrational. In this case, the Court of Appeals believed that by not considering the funding as an element of coverage, there was uniformity amongst all of the patients that made inconsistencies irrational. Other judicial cases also supported patient’s rights to treatment as in the case of *R (on the application of Otley)* vs. *Barking and Dagenham NHS Primary Care Trust* of 2007) [49]. Otley suffered from colorectal cancer and she was denied treatment coverage. The Court ruled that there were no other potential drug treatments and the clinical trial results were not exhaustive to preclude the potential for drug effectiveness in her case since the drug effectiveness was based on the clinical trial for other patients in her patient cohort.

## Discussion

Rare disease patients and their families encounter health and financial barriers in the availability of diagnostic, prevention and treatment alternatives, coverage for existing care, general awareness in society and the healthcare system about rare diseases, and access to educational resources for their own comprehension of their condition. In spite of the increase in approvals, marketed orphan drugs address only a fraction of the large number of rare diseases and conditions that affect millions of patients’ worldwide showing that the unmet needs of the rare disease patient population remain a challenge. Treatment alternatives for rare diseases are limited and health inequities are persistent; some rare diseases have several therapeutic alternatives approved and marketed; whereas, for other rare diseases, there is currently little, if any, research or investment into potential orphan drugs.

In the study period, the diseases with the highest number of FDA orphan drug approvals were hemophilia (including all types of A and B) and acute lymphoblastic leukemia. Cancer is increasingly targeted for orphan drug approvals [17, 35, 50]. This could be a trend towards more stratified medicine [35] or an attempt by the industry to utilize ODA incentives to approve oncology drugs for orphan drug indications that once approved, are often used off-label [51].

Aligning pharmaceutical company incentives with societal budgetary constraints is necessary. Orphan drugs can be profitable even in rare diseases with very small number of patients. The increased number of orphan drug patients and faster than inflation growth in costly orphan drugs pose an economic burden for patients, public programs and private third party payers. Still, the allocation of resources to balance societal priorities has not being addressed in a comprehensive way.

Despite the legislative evidence that there is a commitment to provide a level of care to individuals with rare diseases, there is not a comprehensive moral or ethical justification for the allocation of these resources codified in our societal context. Issues of distributive justice and egalitarian principles of equitable healthcare provide some basis to treat these conditions, but as the number of conditions and treatment cost continue to grow, there are no clear ethical mandates related to how to address the long-term problem.

At the nexus of this complex regulatory, ethical, economic and clinical issue, it remains unresolved the economic incentives and ethical imperatives trade-offs in strengthening access to safe, effective and affordable treatments for patients with rare diseases. The dearth of information on the patients’ health status and overall costs of rare diseases prior to patients gaining access to orphan drugs makes it challenging to assess the cost-effectiveness of orphan drugs. Thus, balancing the cost of economic incentives for the development of orphan drugs against the overall benefits and improvements in health outcomes remains of critical importance [17, 23, 33, 40, 52]. Otherwise, there is a risk of aggravating market failures and perpetuating inefficiencies.

The literature has interrogated questions at the periphery of this issue and assessed the merits of the Orphan Drug Act of 1983. A robust analysis of the policy outcomes has yet to be completed, namely, there is need for empirical evidence of the orphan drug treatment costs, its effectiveness and the long-term benefits for patients and the society at large. On the other hand, the concerns related to innovation of products and equating incentives for pharmaceutical companies to the societal value of orphan drugs will continue to be a daunting policy challenge. Even if a society could agree on the societal value of orphan drugs, there are additional patient needs and treatment costs associated with individuals reaching certain, accepted upon, health status beyond the cost of a drug.

Study findings must be interpreted with caution given the heterogeneity of the drugs approved for orphan indications. Orphan designations are granted to a large number of drugs that treat rare diseases and conditions that may be very different by nature. The cost of treatment and clinical outcomes may also vary considerably across and within rare diseases. Despite these challenges orphan drugs often represent the only hope for patients and their families. Thus, the development and marketing of safe and effective treatments remains critical to address the health needs of the rare disease community.

## Conclusion

It is often difficult for those in the rare disease community to not trumpet the success of the ODA, based on the increase in the orphan drug designations and FDA approvals after 1983. However, over 30 years later, we are still using the benchmark of a 20^th^ century framework to measure our success. Advocates are reluctant to point out any flaws based on the fear of stifling innovation, but with over 6,500 diseases needing treatment, there is evidence to suggest patients’ unmet health needs remains a concern and more effective incentives have to be implemented. There is an ethical imperative of addressing patients with rare diseases access to orphan drugs.
